# Explainable machine learning for the prediction of Alzheimer’s disease-related cognitive impairment: a consensus feature selection approach

**DOI:** 10.1186/s12911-026-03585-z

**Published:** 2026-05-29

**Authors:** Fulden Cantaş Türkiş

**Affiliations:** Division of Biostatistics, Faculty of Medicine, Muğla Sıtkı Koçman University, Muğla, 48000 Turkey

**Keywords:** Feature selection, Machine learning, Explainability, Calibration, Clinical utility

## Abstract

**Background:**

Early identification of Alzheimer’s disease-related cognitive impairment remains challenging, and existing machine learning (ML) models often suffer from feature instability and limited interpretability. This study developed robust and explainable ML models using cerebrospinal fluid (CSF) biomarkers by systematically comparing sparsity-based (LASSO), importance-based (Boruta), and consensus feature selection strategies.

**Methods:**

A publicly available cohort of 333 individuals (91 cognitively impaired, 242 cognitively normal) was analyzed. Data were split into training (70%) and independent test (30%) sets. Multiple classifiers, including Elastic Net-regularized logistic regression (LR), support vector machine (SVM), random forest, XGBoost, and Naive Bayes (NB), were trained using repeated 5-fold cross-validation (10 repetitions; 10 × 5-fold cross-validation) with class weighting. Model performance was evaluated using discrimination, calibration, and clinical utility metrics, and interpretability was assessed using SHAP.

**Results:**

All models demonstrated strong discriminative performance on the test set (AUROC 0.861–0.958). LASSO-based models showed high specificity, Boruta-based models achieved higher sensitivity, and consensus-based models provided the most balanced performance. The consensus-LR and -SVM models achieved AUROC values of 0.954 and 0.951, respectively. Beyond discrimination, the consensus-LR model demonstrated good calibration and consistent net clinical benefit in decision curve analysis, analyses that remain relatively underreported in the Alzheimer’s disease machine learning literature. SHAP analyses highlighted biologically plausible contributions from key biomarkers, including tau, Aβ42, NT-proBNP, pancreatic polypeptide, and IL-7.

**Conclusions:**

In summary, stable and interpretable ML models for Alzheimer’s disease-related cognitive impairment can be developed using CSF-derived biomarkers obtained through lumbar puncture. The proposed consensus-based feature selection framework improves feature stability and model transparency, facilitating the discrimination between cognitively normal and impaired individuals and providing a foundation for future external validation studies.

**Supplementary Information:**

The online version contains supplementary material available at 10.1186/s12911-026-03585-z.

## Background

Alzheimer’s disease (AD) is a progressive neurodegenerative disorder characterized by a prolonged preclinical phase during which pathological changes accumulate years before the onset of clinical symptoms. Early identification of individuals with potential cognitive impairment remains a major challenge in clinical practice. In this context, machine learning (ML) and artificial intelligence (AI) have gained substantial attention due to their capacity to model complex, nonlinear relationships in high-dimensional biomedical data, offering potential advantages over traditional statistical approaches for the diagnosis and prognosis of AD [1–4].

A wide spectrum of ML-based models has been proposed for AD detection, integrating heterogeneous data sources such as neuroimaging, cerebrospinal fluid (CSF) biomarkers, blood-based biomarkers, and clinical assessments [2, 4, 5]. Recent reviews and systematic analyses have documented rapid methodological advances and promising predictive performance; however, they have also emphasized persistent challenges related to robustness, reproducibility, and clinical translation [3, 5]. In particular, high performance reported in single-cohort studies often fails to generalize to independent populations, raising concerns about overfitting and limited external validity [6, 7].

Biomarker-driven ML models constitute a central component of contemporary AD research. Established CSF biomarkers, including amyloid-β and tau proteins, provide important insights into disease pathology and are integral to the ATN framework [8]. Nevertheless, increasing evidence suggests that single-biomarker approaches and fixed cut-off-based strategies inadequately capture the biological heterogeneity of AD [9–12]. Multivariate ML-based analyses have demonstrated that continuous biomarker profiles and combined biomarker representations offer more informative and flexible characterizations of disease status and progression [9, 11, 12]. Despite these advances, the selection of relevant biomarkers remains a critical and unresolved issue.

A key but underexplored limitation of existing ML studies lies in the instability of feature selection procedures. Different feature selection strategies-such as sparsity-driven embedded methods and importance-based wrapper approaches-often yield divergent biomarker subsets, even when applied to the same dataset. This variability contributes to inconsistent model behavior across cohorts and complicates biological interpretation, thereby limiting clinical confidence and translational value. Importantly, most prior studies rely on a single feature selection technique, without systematically evaluating how different selection paradigms influence model stability, interpretability, and clinical relevance [13, 14]. Consequently, many explainable ML studies in Alzheimer’s disease continue to depend on a single feature selection strategy, which may produce unstable biomarker subsets and reduce reproducibility across datasets. This limitation highlights the need for approaches that integrate complementary feature selection paradigms to identify more stable and biologically meaningful predictors. In addition, Alzheimer’s disease datasets frequently exhibit pronounced class imbalance, with substantially larger cognitively normal groups compared to impaired cases, further challenging model training and evaluation.

In parallel, the interpretability of ML models has emerged as a critical requirement for clinical adoption. While deep learning and ensemble methods frequently achieve high predictive accuracy, their black-box nature hinders clinical trust. Explainable AI (XAI) techniques, such as SHAP-based feature attribution and interpretable model architectures, have been proposed not only to enhance transparency but also to support biological validation by revealing how individual biomarkers and their interactions contribute to model predictions [15–19]. When combined with robust feature selection, XAI offers a pathway toward models that are both accurate and clinically meaningful.

Against this background, the present study addresses two interconnected gaps in the existing literature. First, it systematically evaluates the impact of different feature selection paradigms by comparing a sparsity-driven approach (LASSO), an importance-driven all-relevant method (Boruta), and a consensus-based feature selection strategy that identifies predictors consistently selected across methods. Second, it integrates XAI techniques to assess the stability, biological plausibility, and clinical interpretability of the resulting classification models. Using a well-characterized open-access cohort originally reported by Craig-Schapiro et al. [20], this study provides a unified and reproducible modeling framework that jointly evaluates feature stability, model explainability, and clinical applicability, thereby contributing to the development of robust and interpretable machine learning models for Alzheimer’s disease-related cognitive impairment.

## Methods

**Fig. 1:**
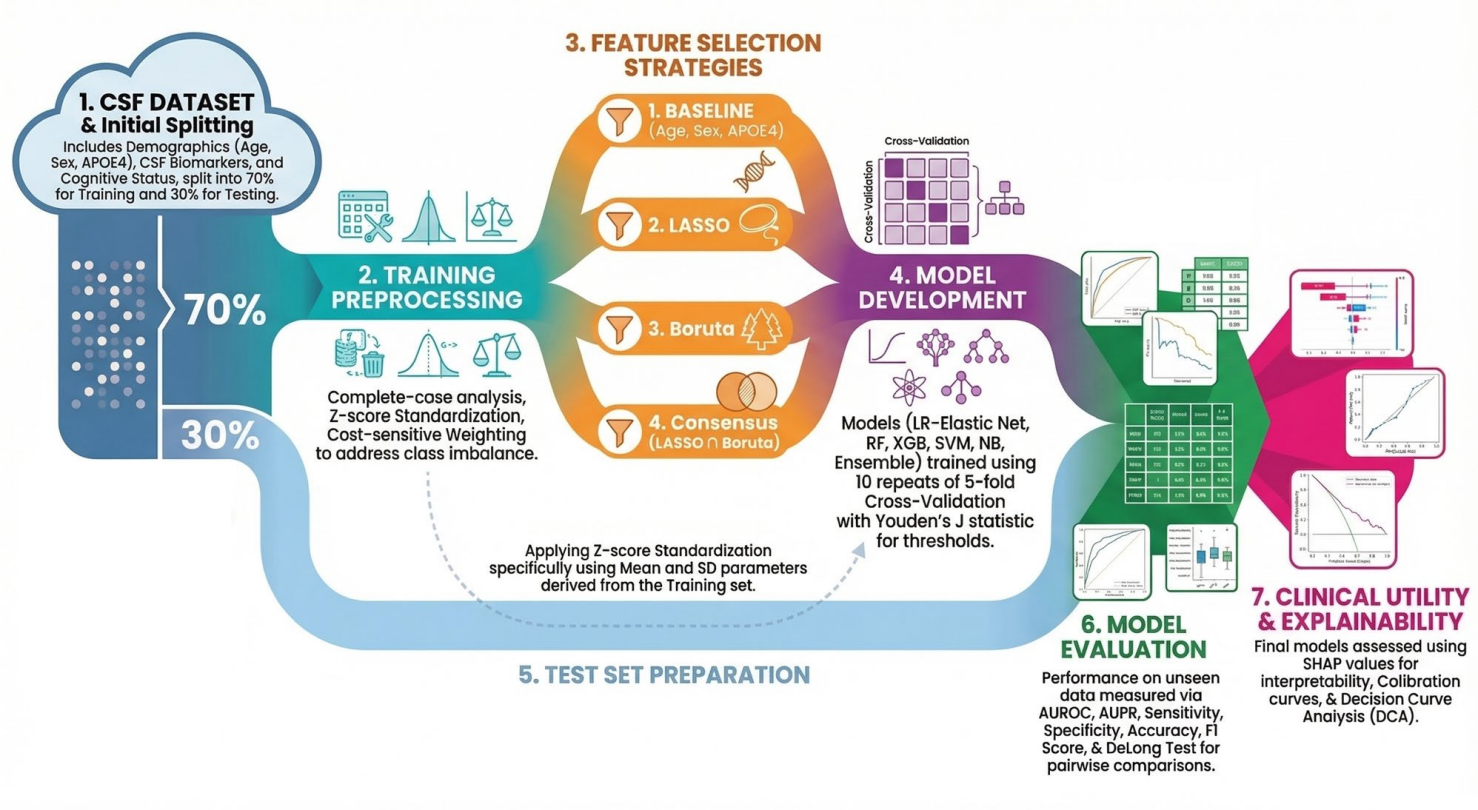
Schematic overview of the machine learning workflow, including data preprocessing, feature selection, model development, validation, and explainability analyses

The overall machine learning workflow, including data splitting, preprocessing, feature selection, model development, validation, and explainability analyses, is summarized in Fig. 1.

### Study design and setting

This study is a secondary analysis of a publicly available dataset containing CSF biomarkers and demographic information for individuals classified as cognitively normal or impaired. The original data were collected as part of a previously published cohort study originally reported by Craig-Schapiro et al. [20]. Our objective was to develop and compare machine-learning models to discriminate impaired individuals from controls using CSF biomarker profiles. All analyses were conducted independently of the original study.

### Participants and data splitting

All individuals with complete CSF biomarker measurements and a valid cognitive status label were included. Cognitive status was defined based on the Clinical Dementia Rating (CDR), where CDR = 0 indicated a control and CDR > 0 indicated an impaired participant, consistent with the grouping strategy used in the original study and subsequent machine learning applications of the same dataset [20, 21]. No additional exclusion criteria were applied. The dataset consisted of all eligible participants from the original cohort. For model development, the data were divided into a training and a testing set using a 70:30 stratified split to preserve the original class distribution. This approach was chosen to ensure the availability of an independent hold-out test set for final model evaluation, thereby enabling an unbiased assessment of model performance on previously unseen data. In addition, maintaining a separate test set allowed the evaluation of discrimination, calibration, and clinical utility metrics without introducing information leakage during model development.

### Variables

The primary outcome was cognitive status, coded as 0 (control) and 1 (impaired). This variable was used as the binary dependent variable in all models. Predictors included a wide panel of CSF biomarkers, age, sex (male = 1, female = 0), and APOE4 genotype status (carrier = 1, non-carrier = 0). Only measured biomarkers and demographic variables were used; no derived variables were created. Age, sex, and APOE4 status were treated as potential confounders and were included in the feature selection process alongside the biomarker variables.

### Data preprocessing

The dataset used in this study did not contain missing values for the included variables. Therefore, complete-case analysis was performed. Because biomarker measurements were reported on different scales, Z-score standardization was applied to all biomarker variables. Standardization parameters (mean and standard deviation) were computed using the training set only and then applied to the testing set to avoid information leakage. Sex, APOE4 genotype, and the outcome variable were not standardized, and the binary predictors were retained in their original numeric coding (0/1).

### Addressing potential sources of bias

#### Class imbalance

Imbalance between impaired and control participants was addressed using cost-sensitive weighting. Class weights for the minority class were defined as the ratio of the number of majority-class observations to the number of minority-class observations in the training set, resulting in a higher penalty for misclassifying impaired cases than controls.

#### Overfitting prevention

To minimize overfitting, a 10 × 5-fold cross-validation, which reflected a balance between computational feasibility and the need for stable performance estimates in a moderately sized and class-imbalanced dataset, was used for all model development and hyperparameter tuning. The same cross-validation folds were applied to all algorithms to ensure fair comparison.

#### Prevention of information leakage

No information from the testing set was used during preprocessing, feature selection, model training, or threshold determination. All thresholds were chosen exclusively from training predictions.

### Feature selection

#### LASSO-regularized feature selection

LASSO regression [22] was employed solely for feature selection using the standardized training data. Feature selection was performed on the training dataset prior to model development. The shrinkage parameter (λ) was optimized via internal 10-fold cross-validation based on the minimum binomial deviance criterion (λ_min). The optimal value was λ_min = 0.0346, corresponding to − log(λ) ≈ 3.36 on the cross-validation curve. Predictors with non-zero coefficients at λ_min were retained and defined as the LASSO-selected feature set for subsequent ML analyses. The selected features were then used consistently in all subsequent model training and evaluation steps. Model coefficients were not interpreted at this stage, as LASSO was used exclusively for variable screening rather than effect estimation.

#### Boruta-based feature selection

The Boruta algorithm was employed as a complementary all-relevant feature selection approach to identify predictors with evidence of relevance to the outcome. Feature relevance was determined by iteratively comparing the importance of original variables with that of randomized shadow attributes within a Random Forest framework [23]. The TentativeRoughFix procedure was subsequently applied to resolve tentative features, yielding the final Boruta-confirmed feature set for subsequent ML analyses.

#### Consensus feature selection (LASSO ∩ Boruta)

To assess the stability of the selected predictors and to identify features consistently supported by both selection procedures, we constructed a single combined feature set representing the intersection of LASSO-selected and Boruta-confirmed variables. This intersection set included only predictors jointly identified as relevant by both methods and was used to evaluate whether models trained on a more conservative and agreement-based subset of biomarkers demonstrated improved robustness or interpretability.

### Model development, optimization, and evaluation

All models were trained within a 10 × 5-fold cross-validation framework, in which the area under the receiver operating characteristic curve (AUROC) [24] was used as the primary optimization metric. Within this framework, several ML algorithms were developed and evaluated, including Elastic Net-regularized logistic regression (LR) [25, 26], Random Forest (RF) [27], Extreme Gradient Boosting (XGBoost) [28], Support Vector Machines (SVM) with a radial basis function (RBF) kernel [29], and a Naive Bayes (NB) [30] classifier. Each algorithm was tuned using predefined, model-specific hyperparameter grids, and observation-level class-weight adjustments derived from the imbalance correction procedure were incorporated when supported by the respective modeling approach. The final optimized hyperparameter configurations for all models and feature selection strategies are provided in Supplementary Table S1. This strategy ensured that all models were trained, optimized, and evaluated under consistent, comparable, and fully reproducible conditions. In addition, multicollinearity among predictors was assessed using variance inflation factors (VIF) calculated from a standard (unpenalized) logistic regression model.

In an additional exploratory analysis, a weighted soft-voting ensemble model was constructed using the best-performing classifiers within the consensus feature set (LR, SVM, and XGBoost). Model weights were determined based on AUROC obtained from the training data. Ensemble probabilities were computed as a weighted average of individual model predictions, and the optimal decision threshold was derived from the training set using Youden’s J statistic to avoid information leakage.

### Threshold selection and model evaluation

Optimal decision thresholds were identified using Youden’s J statistic derived from cross-validated predictions in the training set, and these thresholds were subsequently applied to the untouched testing set to avoid information leakage. Model performance on the testing data was assessed using a comprehensive set of metrics, including AUROC, the area under the precision-recall curve (AUPR), accuracy, sensitivity, specificity, precision, F1 score, Brier score, and the Matthews correlation coefficient (MCC). Furthermore, ROC curves and their corresponding 95% confidence intervals were generated to enable a consistent and rigorous comparison of discriminative performance across all models. Pairwise comparisons of AUROC values between models were conducted using DeLong’s test [31].

### Explainability analysis

Model interpretability was assessed using Kernel SHAP [18]. Both global and local explanations were generated to quantify the contribution of individual predictors to the model’s output. At the global level, SHAP beeswarm plots were employed to visualize the distribution, magnitude, and direction of feature contributions across all individuals, allowing simultaneous assessment of inter-individual variability and the relationship between feature values and their corresponding SHAP effects. In addition, local SHAP explanations were produced for two representative cases-one cognitively impaired and one cognitively normal-to illustrate individual-level feature contributions and to enhance transparency of model decision-making. In an additional analysis, the consistency between coefficient-based and SHAP-based interpretations was evaluated by comparing the absolute values of the logistic regression coefficients with the mean absolute SHAP values across features. The strength of the association was quantified using Spearman’s rank correlation coefficient. In addition to the consensus-LR model, SHAP analyses were also conducted for all black-box models (RF, XGBoost, SVM, and NB) based on the consensus feature set, to evaluate the consistency of feature attribution patterns across different model architectures.

### Computational environment and reproducibility

All analyses were conducted using R software (version 4.5.1). The modeling pipeline was implemented using the *caret*, *glmnet*, *Boruta*, *xgboost*, *iml*, *pROC*, *PRROC*, and *rmda* packages [32–36]. To ensure reproducibility, a fixed random seed was applied consistently across all preprocessing, feature selection, model training, and evaluation steps. In addition, all preprocessing, feature selection, model training, and evaluation procedures were implemented within a fully reproducible and seed-controlled pipeline, with consistent cross-validation folds across all models to ensure methodological transparency and comparability. All analysis scripts can be made available upon reasonable request.

### Model calibration

Model calibration was evaluated to assess the agreement between predicted probabilities and proportion of observed outcomes. Calibration performance was examined using loess-smoothed calibration curves with 95% confidence intervals generated via bootstrapping. Calibration plots were constructed based on predictions from the independent test set, accompanied by rug plots to illustrate the distribution of predicted probabilities [37]. The Brier score was additionally reported as a global measure of probabilistic accuracy.

### Decision curve analysis

Decision curve analysis (DCA) [38] was performed to evaluate the potential clinical utility of the consensus-LR model. Net benefit was calculated across a range of threshold probabilities by comparing the model-based strategy with treat-all and treat-none strategies. All decision curve analyses were conducted on the independent test set.

## Results

### Study population and dataset characteristics

The initial dataset comprised 333 participants (91 [27.3%] cognitively impaired and 242 [72.7%] cognitively normal), with 130 candidate predictors including CSF biomarkers as well as demographic and genetic variables. The dataset exhibited a moderate class imbalance, with cognitively normal participants representing the majority class.

Baseline characteristics stratified by cognitive status are presented in Table 1. Compared with cognitively normal individuals, the impaired group was older (*p* < 0.001), had a lower proportion of females (*p* = 0.027), and a higher prevalence of APOE4 carriers (*p* < 0.001). In addition, cerebrospinal fluid biomarker profiles differed between groups, with higher tau and p-tau levels and lower Aβ42 levels observed in cognitively impaired participants (all *p* < 0.001).

**Table 1:** Baseline characteristics and cerebrospinal fluid biomarker profiles stratified by cognitive status

Characteristic	Cognitive status		*p*
	Normal (*n* = 242)	Impaired (*n* = 91)	
Age, years	71.60 (7.40)	75.28 (7.02)	**< 0.001**
Sex			**0.027**
Female	157 (64.9)	47 (51.6)	
Male	85 (35.1)	44 (48.4)	
APOE4 status			**< 0.001**
Non-carrier	165 (68.2)	41 (45.1)	
Carrier	77 (31.8)	50 (54.9)	
Tau (pg/mL)	314.80 (168.88)	549.96 (272.80)	**< 0.001**
p-tau (pg/mL)	56.32 (25.16)	82.98 (42.76)	**< 0.001**
Aβ42 (pg/mL)	606.90 (234.13)	411.18 (211.53)	**< 0.001**

Values are presented as mean (standard deviation) for continuous variables and number (percentage) for categorical variables

APOE4: apolipoprotein E4 allele; tau: total tau protein; p-tau: phosphorylated tau; Aβ42: amyloid-beta 42

### Variable selection using LASSO

**Fig. 2:**
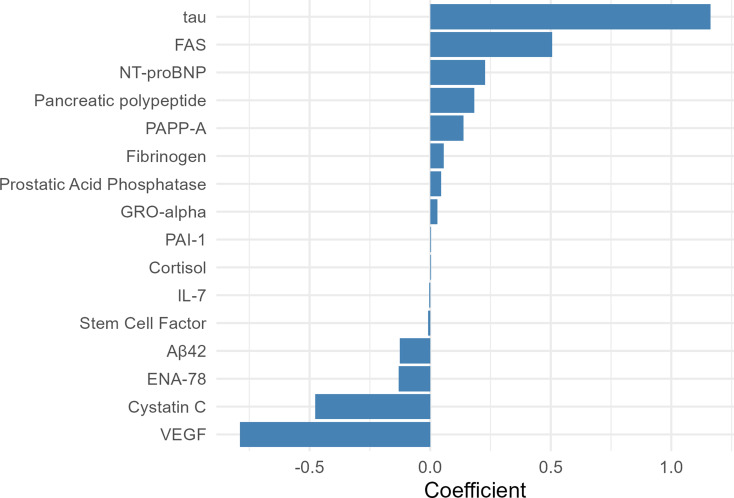
LASSO-based feature selection results

Using a LASSO-based feature selection approach, a subset of predictors with non-zero coefficients was identified from the high-dimensional biomarker space. At the optimal penalty parameter identified via cross-validation, a total of 16 variables were retained, encompassing both established Alzheimer’s disease-related biomarkers and additional protein candidates. As shown in Fig. 2, the selected features included tau, FAS, NT-proBNP, pancreatic polypeptide, PAPP-A, fibrinogen, prostatic acid phosphatase, GRO-alpha, PAI-1, Cortisol, IL-7, stem cell factor, Aβ42, ENA-78, cystatin C, and VEGF. Figure 2 displays the non-zero coefficients of the retained variables, providing a concise overview of the sparse predictor set selected by the LASSO model.

### Variable selection using Boruta

**Fig. 3:**
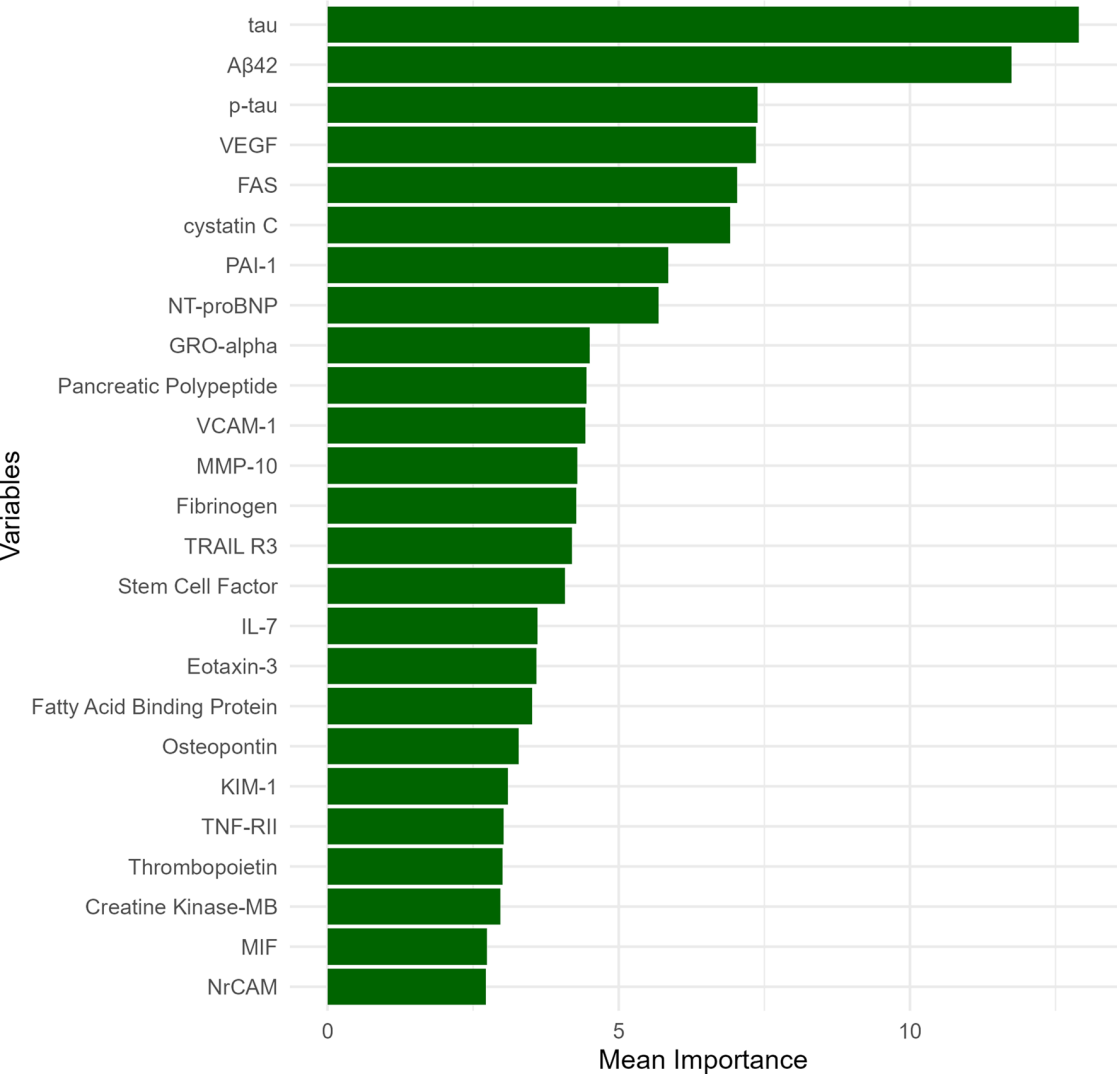
Importance of confirmed biomarkers identified by the Boruta feature selection algorithm. Bars represent the mean importance values derived from the Boruta procedure based on random forest importance measures. Higher importance scores indicate stronger contributions of the corresponding biomarkers to the classification model

Using the Boruta all-relevant feature selection approach, a broader subset of predictors was identified based on their importance relative to shadow attributes. Following resolution of tentative features via the TentativeRoughFix procedure, 25 variables were confirmed as relevant and retained for subsequent analyses. As shown in Fig. 3, the Boruta-confirmed feature set included NrCAM, MIF, creatine kinase-MB, thrombopoietin, TNF-RII, KIM-1, osteopontin, fatty acid binding protein, eotaxin-3, IL-7, stem cell factor, TRAIL R3, fibrinogen, MMP-10, VCAM-1, pancreatic polypeptide, GRO-alpha, NT-proBNP, PAI-1, cystatin C, FAS, VEGF, p-tau, Aβ42, and tau. Figure 3 illustrates the importance ranking of features confirmed by the Boruta feature selection algorithm.

### Consensus feature set derived from LASSO and Boruta

A consensus feature set was derived by intersecting the variables selected by the LASSO and Boruta feature selection procedures. This intersection yielded a reduced subset of predictors simultaneously retained by both methods, representing biomarkers consistently supported across distinct selection frameworks.

The consensus feature set comprised IL-7, pancreatic polypeptide, stem cell factor, PAI-1, NT-proBNP, Aβ42, tau, FAS, fibrinogen, GRO-alpha, VEGF, and cystatin C. This consensus-based subset was subsequently used for model development and comparative performance evaluation alongside the LASSO- and Boruta-derived feature sets.

### Model performance across feature sets

The predictive performance of all models, including the demographic-only baseline and biomarker-based models across different feature selection strategies, is summarized in Table 2.

As a baseline comparison, models incorporating only demographic variables (age, sex, and APOE4 status) were evaluated. These demographic-only models demonstrated modest discriminative performance, with AUROC values ranging from 0.708 to 0.757 across classifiers. Although these models achieved reasonable specificity, their overall predictive performance remained substantially lower than models incorporating CSF biomarkers.

The predictive performance of ML models was further evaluated across three feature selection strategies: LASSO-selected, Boruta-selected, and consensus (LASSO ∩ Boruta) feature sets. For each feature set, LR, RF, XGBoost, SVM, and NB classifiers were trained and evaluated on the independent test set using a unified performance assessment framework.

Models trained on the LASSO-selected feature set demonstrated consistently high specificity across all classifiers, exceeding 0.900 in each case. Discriminative performance was strong, with AUROC values ranging from 0.914 to 0.958. Among these models, SVM and LR achieved the highest AUROC values, whereas sensitivity remained comparatively lower, indicating a conservative classification tendency favoring the negative class.

For the Boruta-selected feature set, models generally exhibited higher sensitivity compared with those trained on LASSO-selected features. Sensitivity exceeded 0.800 for both LR and SVM classifiers, accompanied by balanced accuracy and robust discriminative performance. Overall calibration was satisfactory, as reflected by favorable Brier scores across classifiers.

Models developed using the consensus feature set showed stable and well-balanced performance across multiple evaluation metrics. Within the consensus feature set, all included predictors exhibited variance inflation factor (VIF) values below 5 (range: 1.31–3.99), indicating no substantial multicollinearity. These models achieved high specificity (≥ 0.904) alongside moderate-to-high sensitivity, resulting in favorable F1 scores and MCCs. Discriminative ability remained robust, with AUROC values consistently above 0.915 and the highest AUPR values observed for LR and SVM classifiers.

**Fig. 4:**
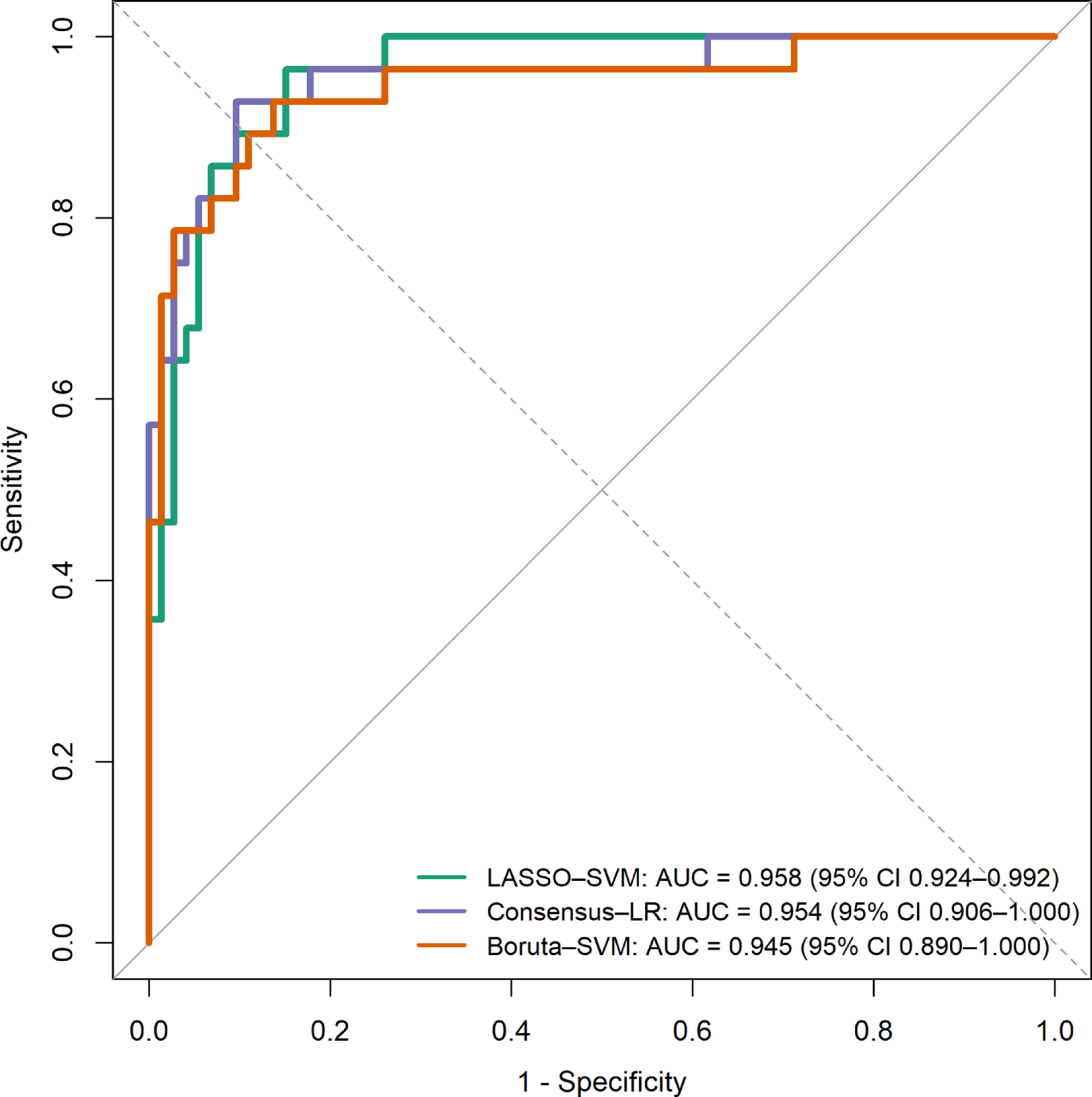
Receiver operating characteristic (ROC) curves of the best-performing models across feature selection strategies

A direct comparison of the best-performing models derived from each feature selection strategy is illustrated in Fig. 4, which presents the ROC curves. Although the LASSO-SVM model achieved the highest AUROC, pairwise comparisons using DeLong’s test did not reveal statistically significant differences among the three models (all *p* > 0.05).

As an additional exploratory analysis, a weighted soft-voting ensemble model combining the consensus-based LR, SVM, and XGBoost classifiers was also evaluated. As shown in Table 2, the ensemble model achieved the highest accuracy (0.911), sensitivity (0.857), F1 score (0.842), and MCC (0.780) among the consensus-based models, with a low Brier score (0.077), indicating strong overall predictive performance. However, its AUROC (0.947) and AUPR (0.901) did not exceed those of the consensus-LR model, and its increased structural complexity reduced interpretability relative to logistic regression.

Although several models demonstrated strong statistical performance, the consensus-LR model may represent the most clinically deployable option because it combines high discriminative ability with good calibration while retaining the relative interpretability of logistic regression, enabling transparent estimation of the probability of cognitive impairment.

**Table 2:** Predictive performance of baseline (demographic-only) and biomarker-based machine learning models across different feature selection strategies evaluated on the independent test set

Feature Set	Classifier	Accuracy	Sensitivity	Specificity	Precision	F1 score	Brier score	MCC	AUROC [95% CI]	AUPR
Baseline	LR	0.703	0.714	0.699	0.476	0.571	0.214	0.375	0.721 [0.610–0.831]	0.505
	RF	0.713	0.679	0.727	0.487	0.567	0.177	0.372	0.732 [0.619–0.844]	0.539
	XGBoost	0.584	0.714	0.534	0.370	0.488	0.209	0.223	0.708 [0.596–0.820]	0.533
	SVM	0.713	0.679	0.726	0.487	0.567	0.190	0.372	**0.757** [0.647–0.867]	0.627
	NB	0.644	0.714	0.616	0.417	0.526	0.173	0.296	0.728 [0.616–0.840]	0.527
LASSO-selected	LR	0.861	0.571	0.973	0.889	0.696	0.083	0.636	0.945 [0.892–0.998]	0.892
	RF	0.822	0.536	0.932	0.750	0.625	0.119	0.525	0.920 [0.867–0.973]	0.793
	XGBoost	0.851	0.571	0.959	0.842	0.681	0.113	0.607	0.914 [0.849–0.979]	0.813
	SVM	0.871	0.643	0.959	0.857	0.735	0.086	0.664	**0.958** [0.924–0.992]	0.892
	NB	0.861	0.750	0.904	0.750	0.750	0.108	0.654	0.931 [0.884–0.978]	0.819
Boruta-selected	LR	0.901	0.821	0.932	0.821	0.821	0.081	0.753	0.936 [0.868-1]	0.891
	RF	0.871	0.643	0.959	0.857	0.735	0.100	0.664	0.922 [0.851–0.993]	0.878
	XGBoost	0.871	0.607	0.973	0.895	0.723	0.106	0.664	0.895 [0.819–0.972]	0.837
	SVM	0.881	0.893	0.877	0.735	0.806	0.076	0.729	**0.945** [0.890-1]	0.907
	NB	0.812	0.786	0.822	0.629	0.698	0.132	0.572	0.861 [0.773–0.949]	0.747
Consensus	LR	0.891	0.679	0.973	0.905	0.776	0.083	0.718	**0.954** [0.906-1]	0.919
	RF	0.822	0.607	0.904	0.708	0.654	0.110	0.538	0.915 [0.846–0.985]	0.828
	XGBoost	0.871	0.679	0.945	0.826	0.745	0.094	0.666	0.915 [0.847–0.983]	0.853
	SVM	0.881	0.750	0.914	0.808	0.778	0.079	0.698	0.951 [0.902–0.999]	0.904
	NB	0.842	0.679	0.904	0.731	0.704	0.107	0.597	0.925 [0.872–0.979]	0.829
	Ensemble	0.911	0.857	0.932	0.828	0.842	0.077	0.780	0.947 [0.893-1]	0.901

LR: Elastic Net-regularized logistic regression; RF: Random Forest; SVM: Support Vector Machine, NB: Naive Bayes, MCC: Matthew’s Correlation Coefficient. The ensemble model represents a weighted soft-voting combination of LR, SVM, and XGBoost classifiers, with weights and decision threshold derived from the training data

### Model explainability using SHAP

**Fig. 5:**
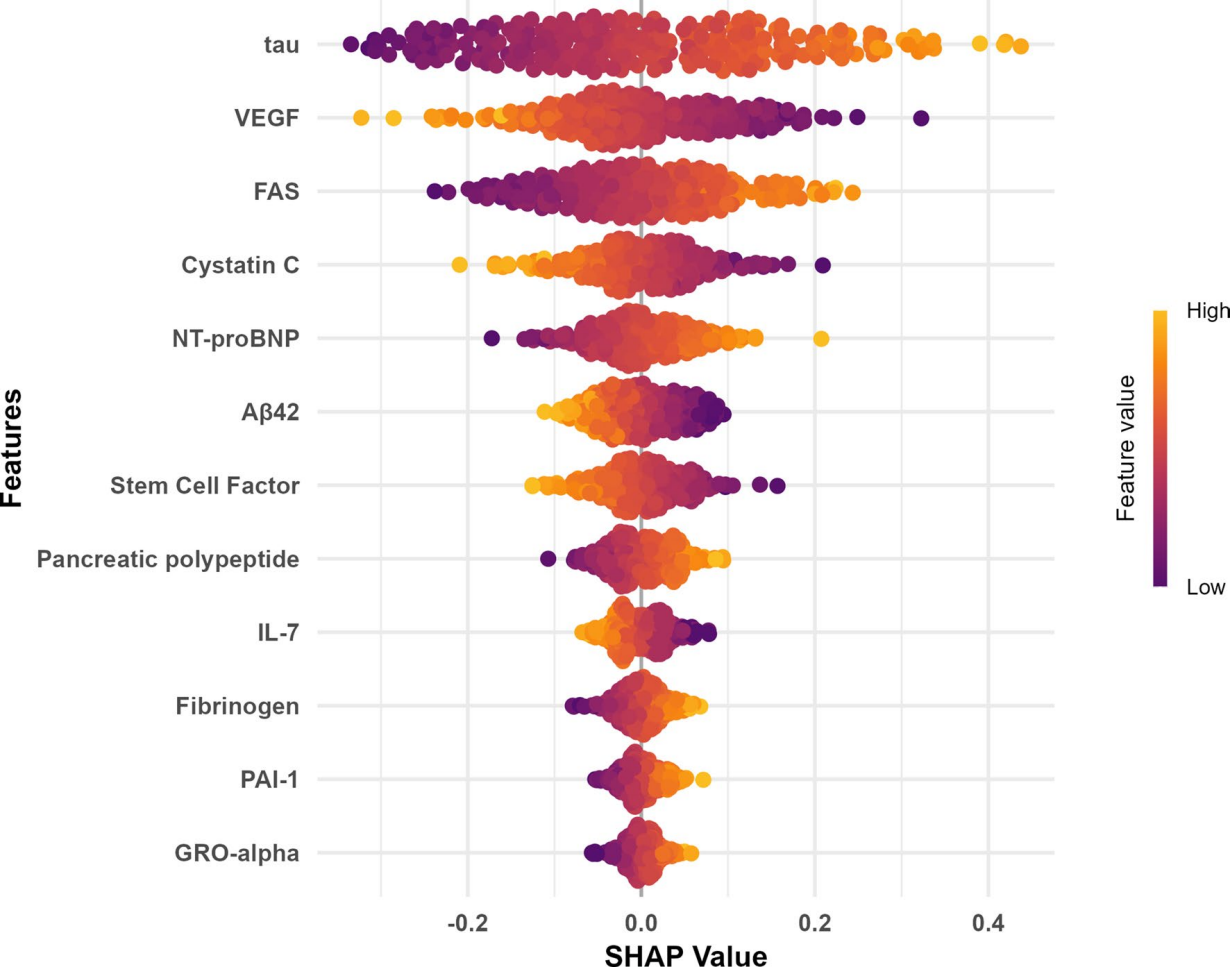
SHAP beeswarm plot of feature contributions to cognitive impairment generated from the independent test set using the consensus-LR model. SHAP beeswarm plot illustrating the distribution of feature contributions across individual subjects in the independent test set for the consensus-LR model. Each point represents a SHAP value for a given feature and individual. The horizontal position indicates the magnitude and direction of the feature’s contribution to the predicted probability of cognitive impairment, where positive values increase the predicted probability and negative values decrease it. Colors represent the corresponding feature values, ranging from low (purple) to high (yellow), enabling visualization of the relationship between feature magnitude and its impact on model predictions

To improve interpretability, SHAP was applied to the consensus-based LR model, which demonstrated robust and well-balanced performance across evaluation metrics. As illustrated in the SHAP beeswarm plot (Fig. 5), tau, VEGF, FAS, cystatin C, and NT-proBNP emerged as the most influential predictors, exhibiting the largest contributions to the model output across individuals. In particular, higher values of tau, FAS, and NT-proBNP were predominantly associated with increased predicted probability of cognitive impairment, whereas higher Aβ42 levels showed a protective pattern. Moreover, VEGF and cystatin C displayed variable contribution patterns across subjects, indicating heterogeneity in their effects, while pancreatic polypeptide and stem cell factor were predominantly associated with positive contributions, with less variability compared to the leading predictors. In contrast, IL-7 was generally associated with lower predicted probability of cognitive impairment, although its overall influence appeared more moderate. Finally, fibrinogen, PAI-1, and GRO-alpha showed relatively small and concentrated SHAP values, suggesting a limited impact on model predictions.

**Fig. 6:**
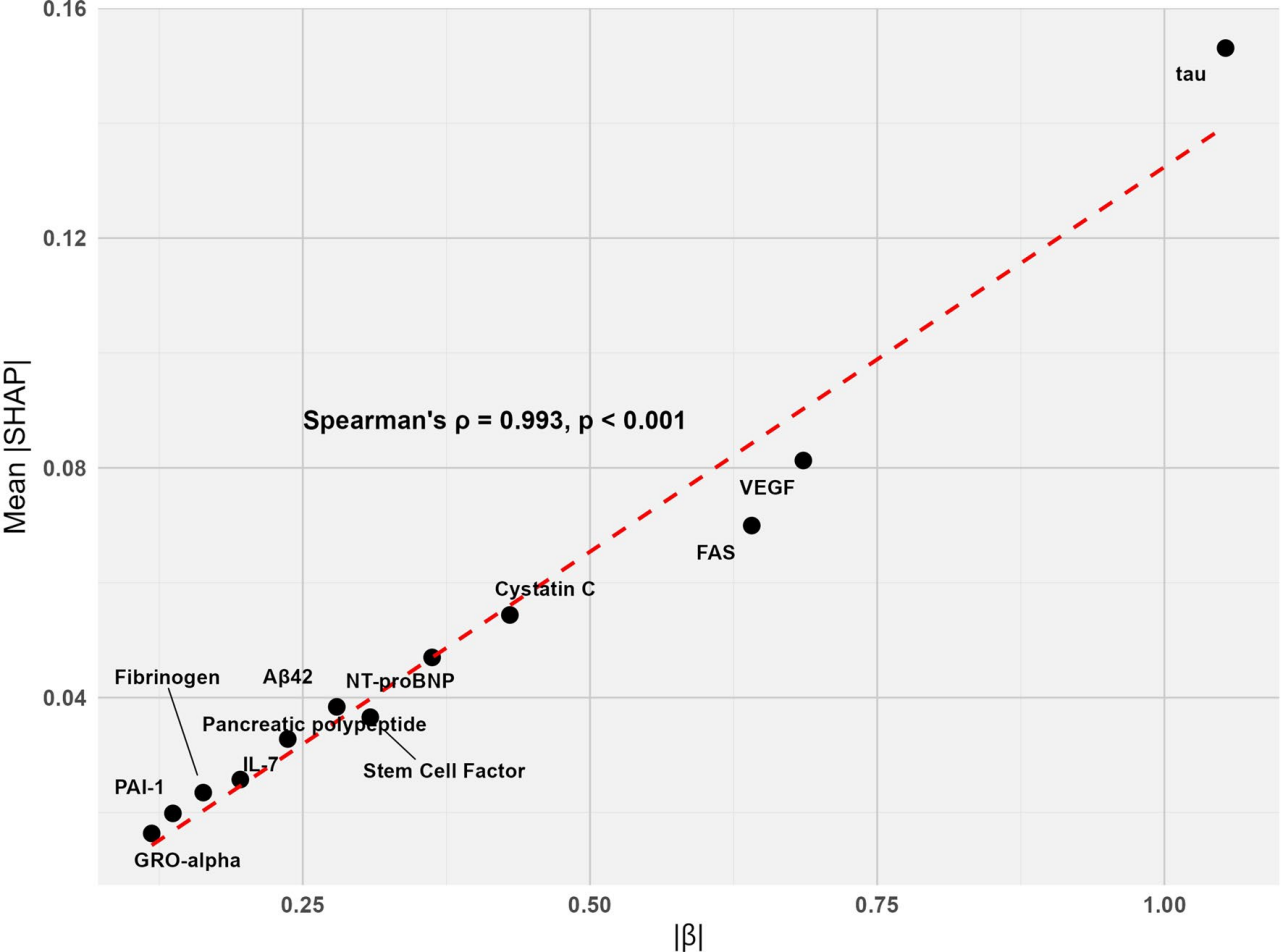
Agreement between logistic regression coefficient magnitude and SHAP-based feature importance in the consensus-LR model. Each point represents a feature included in the consensus-LR model. The x-axis shows the absolute value of the logistic regression coefficient (|β|), reflecting the magnitude of the feature’s effect on the log-odds of cognitive impairment, while the y-axis represents the mean absolute SHAP value, indicating the overall contribution of each feature to model predictions. The dashed red line denotes the fitted linear relationship. A very strong positive correlation (Spearman’s ρ = 0.993, *p* < 0.001) demonstrates a near-perfect agreement in feature importance ranking between coefficient-based and SHAP-based interpretations

To further assess the consistency between coefficient-based and SHAP-based interpretations in the consensus-LR model, the absolute values of the LR coefficients were compared with the mean absolute SHAP values across features (Fig. 6). A very strong positive correlation was observed (Spearman’s ρ = 0.993, *p* < 0.001), indicating a near-perfect concordance in feature importance ranking between the two approaches.

Overall, the SHAP beeswarm plot provided a comprehensive visualization of the distribution and variability of feature effects across individuals, highlighting differences in both the magnitude and direction of contributions.

To further illustrate the model’s decision-making process at the individual level, SHAP explanations were generated for representative impaired and control cases from the test set (Fig. 7a and 7b). For the impaired individual, the predicted probability of impairment (0.602) exceeded the average model prediction (0.383). This increased predicted probability was primarily driven by positive contributions from cystatin C, tau, pancreatic polypeptide, NT-proBNP, and stem cell factor, while FAS, VEGF, and Aβ42 exerted negative contributions that partially counterbalanced the overall prediction. Conversely, for the control individual, the predicted impairment probability was markedly lower than the average prediction (0.052 vs. 0.383), with multiple features-including tau, Aβ42, FAS, VEGF, and IL-7-contributing in a consistently negative direction.

Overall, the SHAP analysis demonstrated that the model integrates multiple biological signals rather than relying on a single dominant predictor. The concordance between global- and individual-level SHAP results indicates a stable and transparent decision structure, supporting the interpretability of the proposed modeling framework.

**Fig. 7a:**
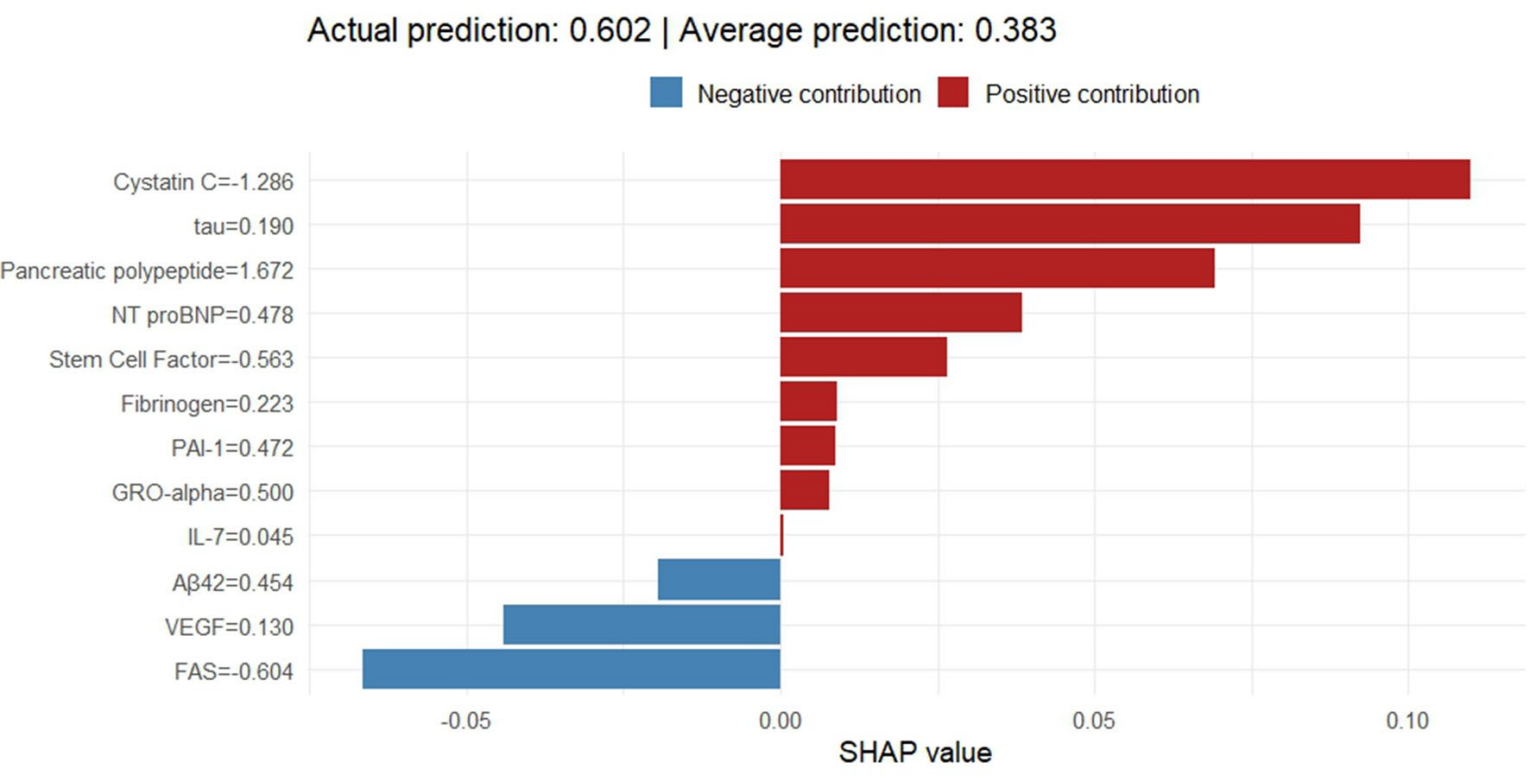
Local SHAP explanation for a representative impaired individual

**Fig. 7b:**
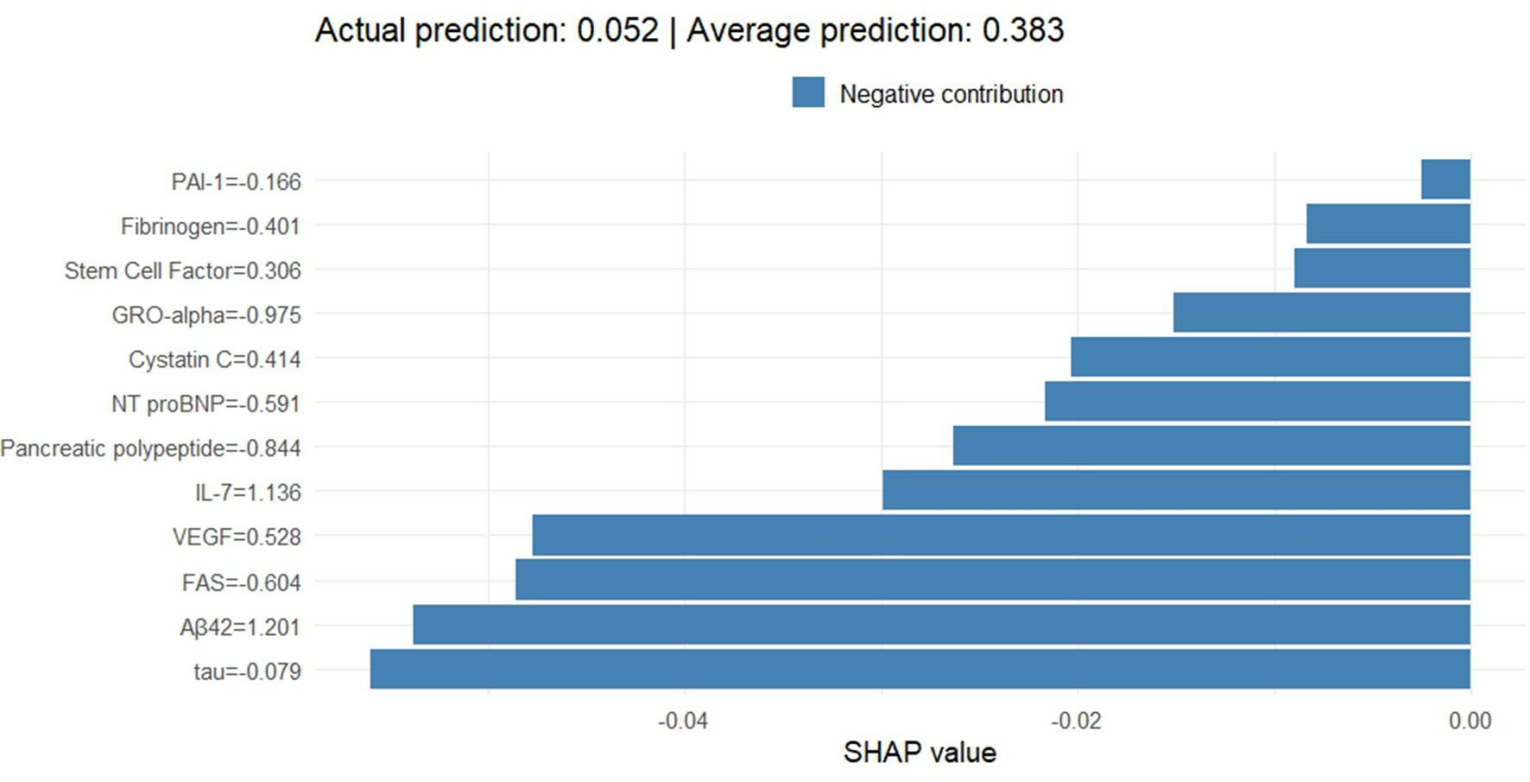
Local SHAP explanation for a representative control individual

To further extend the explainability analysis beyond the inherently interpretable logistic regression model, additional SHAP analyses were performed for the black-box models, including RF, XGBoost, SVM, and NB classifiers. As presented in Supplementary Figure S2, these models demonstrated broadly consistent feature importance patterns, with key biomarkers such as tau, VEGF, and FAS consistently identified as influential predictors, while Aβ42 was also frequently highlighted across models. However, compared with the consensus-LR model, the SHAP distributions of black-box models exhibited greater variability and less consistent directional patterns, particularly for intermediate-ranked features. These findings further support the robustness of the identified predictors while highlighting the enhanced interpretability and stability of the consensus-LR model.

### Model calibration and probability estimation accuracy

**Fig. 8:**
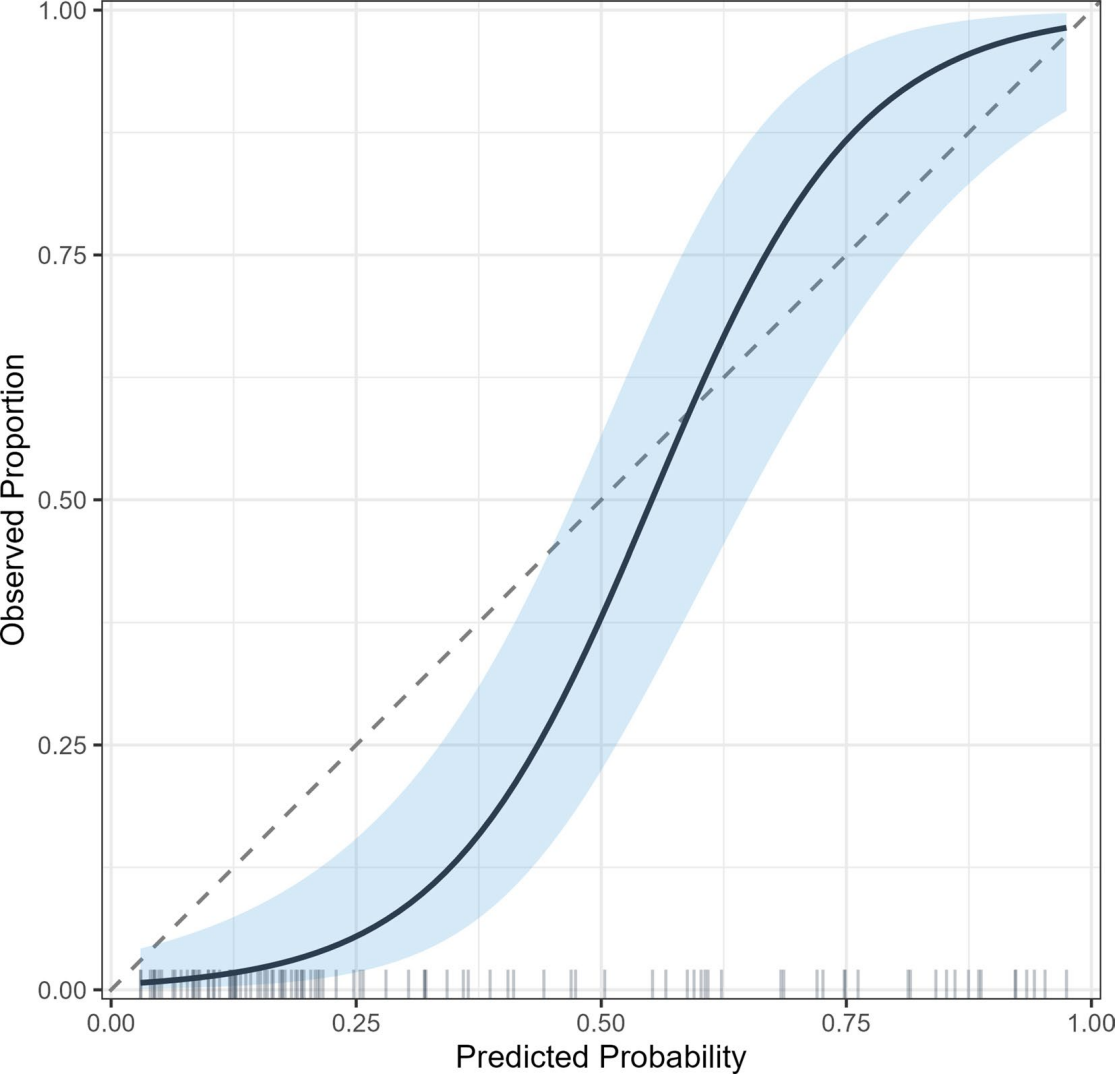
Calibration of the consensus-LR model. Calibration of the consensus-LR model in the independent test set. The plot illustrates the agreement between predicted probabilities and observed outcome proportions. The solid line represents the loess-smoothed calibration curve, with the shaded area indicating the 95% confidence interval. The dashed line corresponds to perfect calibration. Rug marks along the x-axis indicate the distribution of predicted probabilities

To assess the reliability of the model’s predicted probabilities, calibration performance was evaluated using a smoothed calibration curve with 95% confidence intervals, supplemented by a rug plot illustrating data density across predicted probability strata (Fig. 8).

The calibration curve showed close alignment with the ideal diagonal line, representing perfect calibration, across most of the predicted probability range. This suggests good agreement between the model’s predicted probabilities and the observed proportion of cognitively impaired individuals. Minor deviations from the ideal line were observed in the lower (< 0.25) and upper (> 0.75) probability ranges, where data density was lower, as indicated by the rug plot. Within the clinically relevant mid-range of predicted probabilities (approximately 0.25–0.75), where the majority of observations were concentrated, the calibration curve closely followed the diagonal and confidence intervals remained narrow, supporting good calibration.

Calibration analyses performed for the consensus-based alternative models (RF, XGBoost, SVM, NB, and the ensemble model) are presented in Supplementary Figure S1. These models showed greater deviation from the ideal calibration line compared to the consensus-LR model, particularly within the intermediate predicted-probability range. Among them, XGBoost and the ensemble model demonstrated relatively closer agreement with the ideal line, whereas RF, SVM, and NB exhibited greater deviations, indicating reduced reliability in probability estimation despite their strong discriminative performance.

### Decision curve analysis

**Fig. 9:**
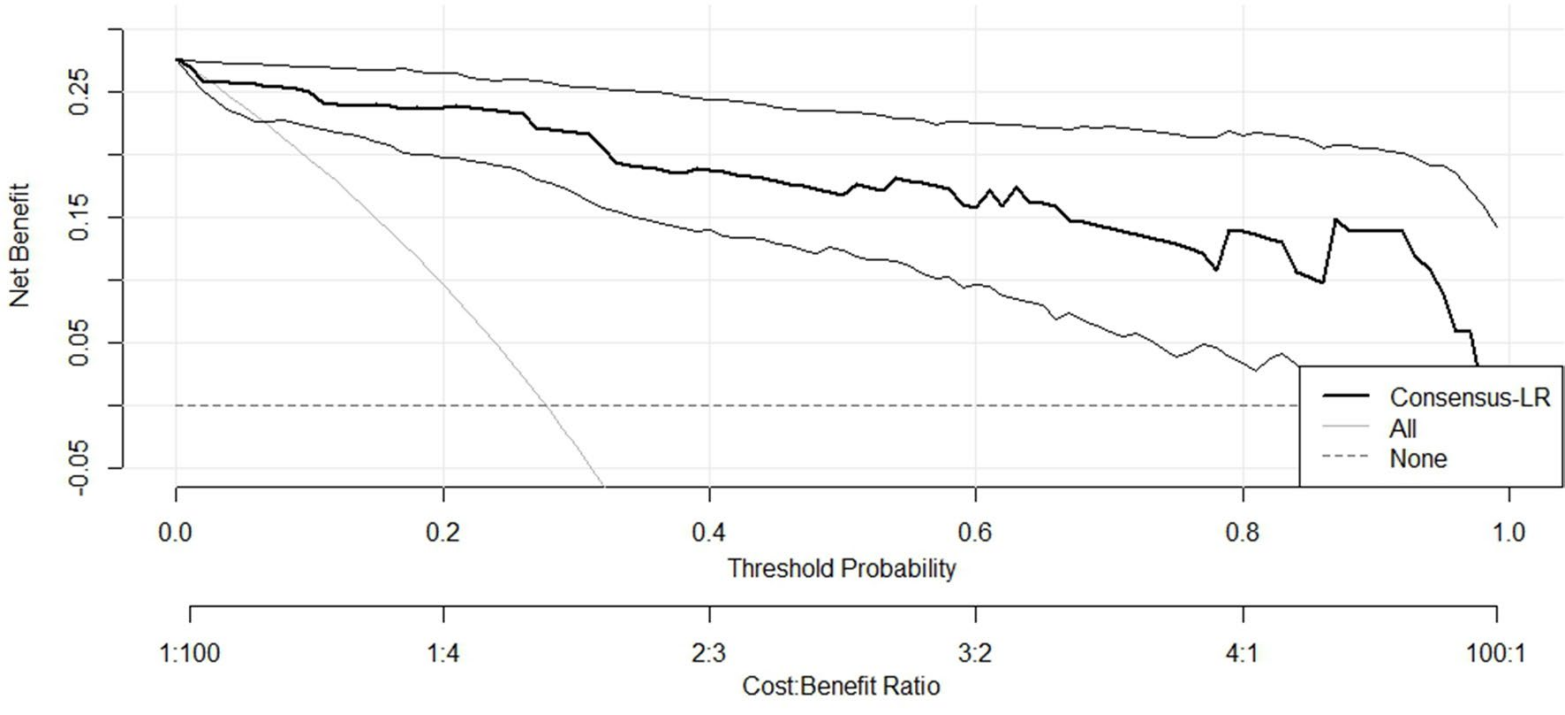
Decision curve analysis of the consensus-LR model. Decision curve analysis illustrating the clinical net benefit of the consensus-LR model across a range of threshold probabilities in the independent test set. The black solid line represents the consensus-LR model, while the light grey solid line indicates the treat-all strategy and the dark grey dashed line represents the treat-none strategy

Across a wide range of clinically relevant threshold probabilities (Fig. 9), the consensus-LR model demonstrated a consistently higher net benefit compared with both the treat-all and treat-none strategies. In particular, within the threshold probability interval of approximately 0.10 to 0.70, the consensus-LR model provided superior net benefit, indicating improved identification of cognitively impaired individuals without a disproportionate increase in false-positive classifications. At threshold probabilities outside this range, particularly at very low and very high thresholds, the model’s net benefit approached or overlapped with that of the default strategies, consistent with clinical scenarios in which either treating all individuals or treating none would be preferred.

## Discussion

This study presents a ML framework for the classification of Alzheimer’s disease**-**related cognitive impairment using clinical variables and CSF-derived biomarkers obtained through lumbar puncture with a particular emphasis on robustness, interpretability, and model transparency. By integrating multiple feature selection strategies and evaluating several ML algorithms, the proposed approach achieved stable and well-balanced discrimination performance on an independent test set. These findings suggest that clinical variables and CSF-derived biomarkers contain meaningful predictive information for distinguishing cognitively normal from cognitively impaired individuals. In clinical contexts, such models may support early identification of individuals who could benefit from closer cognitive monitoring or referral for further diagnostic assessment, rather than serving as a standalone diagnostic instrument.

Importantly, the inclusion of a demographic-only baseline model in the present study provides additional insight into the incremental value of biomarker information. While models based solely on age, sex, and APOE4 status achieved moderate discrimination, their performance was consistently inferior to biomarker-informed models across all evaluation metrics. This comparison suggests that demographic variables alone provide limited discriminative ability, and that the inclusion of CSF-derived biomarkers substantially improves predictive performance. Notably, the overall accuracy of the baseline models remained slightly below the proportion of the majority class (72.7%), which represents the expected accuracy of a naive classifier always predicting the dominant class. This observation is attributable to the imbalanced class distribution and the fact that the baseline models attempt to identify impaired individuals rather than defaulting to majority-class predictions. As a result, improvements in sensitivity were achieved at the expense of overall accuracy. Importantly, this finding highlights that accuracy alone may be misleading in imbalanced classification settings, and reinforces the need to interpret model performance using complementary metrics such as AUROC, sensitivity, specificity, F1 score, and MCC. In this context, the observed AUROC values (0.708–0.757) indicate that demographic variables provide limited but non-negligible discriminative information.

The use of CSF biomarkers to predict a clinically derived outcome such as CDR requires careful interpretation, particularly given the invasive nature of lumbar puncture. However, this approach is consistent with prior studies that have used CSF biomarker profiles to discriminate cognitively impaired individuals defined by CDR status [20, 21]. In this context, CSF biomarkers are not intended to replace clinical evaluation, but to capture underlying biological processes that may precede or complement clinically observable symptoms. As such, modeling the relationship between CSF-derived features and cognitive status can be viewed as an investigation of how neurobiological alterations translate into clinical impairment. This biologically informed perspective may support the identification and categorization of individuals based on cognitive impairment status and enhance understanding of disease mechanisms, particularly in settings where biomarker assessment is already clinically indicated. Nevertheless, the invasive nature of CSF acquisition limits its use in routine screening, and such models should be considered complementary to established clinical assessment pathways.

Beyond these conceptual considerations, an additional methodological strength of the present study is the explicit evaluation of model calibration and clinical utility through decision curve analysis, as well as the benchmarking of biomarker-based models against a demographic-only baseline under a unified modeling framework. While many machine learning studies in Alzheimer’s disease primarily focus on discrimination metrics such as AUROC, fewer studies assess whether predicted probabilities are well calibrated or whether model use would provide net clinical benefit in clinical decision-making. Together, these analyses offer a broader perspective on model performance beyond discrimination alone and may contribute to a more informative assessment of potential clinical applicability.

From a broader perspective, the majority of existing ML studies in Alzheimer’s disease have primarily focused on neuroimaging-derived features, blood-based biomarkers, or CSF-based molecular profiles, often reporting high classification performance under controlled experimental conditions [1, 7, 39]. However, systematic evaluations of this literature have consistently highlighted key limitations, including insufficient external validation, methodological heterogeneity, and limited clinical translation [1]. In this context, the present study contributes to the field by demonstrating that meaningful predictive performance can be achieved using a combination of clinical variables and CSF-derived biomarkers, and by explicitly quantifying the performance gain relative to baseline demographic information, thereby addressing a methodological gap identified in prior reviews.

These findings should also be interpreted in light of the historical evolution of datasets and benchmarks in Alzheimer’s disease research. Early foundational studies established highly standardized cohorts that have since served as reference datasets for a large proportion of ML models [20]. While these datasets have been instrumental in methodological progress, their repeated use has been suggested to raise concerns regarding benchmark dependency and potential overestimation of real-world performance. Models developed using heterogeneous, clinical variables and CSF-derived biomarkers obtained through lumbar puncture, such as the present approach-may therefore offer a complementary perspective for evaluating model performance under more heterogeneous data conditions, which may be relevant when considering potential clinical applicability.

Recent methodological advances have increasingly emphasized multimodal data fusion as a promising direction for prediction tasks in Alzheimer’s disease research [40]. Blood- and CSF-based ML studies further demonstrate that molecular biomarkers, particularly proteomic panels, can achieve high diagnostic accuracy and provide valuable biological insights [10, 11, 41]. Nevertheless, such approaches introduce additional complexity, invasiveness, and resource requirements, which may limit their applicability in routine clinical settings. In this context, the present findings suggest that while demographic information provides a limited baseline, the integration of CSF biomarkers enables a substantial improvement in predictive performance without requiring multimodal data fusion. The performance achieved in the current study using a single, clinically pragmatic data modality suggests that meaningful discrimination between cognitively normal and impaired individuals is feasible even without multimodal integration, and that future extensions may build upon this foundation in a stepwise manner. However, this performance should be interpreted within the context of classification rather than longitudinal risk prediction.

An important conceptual distinction emerging from the literature concerns the definition of “early detection.” Biomarker-driven studies often conceptualize early disease stages in terms of biological pathology, whereas clinically grounded prediction models tend to emphasize early identification of individuals at increased risk of future cognitive decline [8]. This distinction is further demonstrated by prognostic models focusing on conversion from mild cognitive impairment to Alzheimer’s disease using longitudinal multimodal data [42]. While such models provide valuable prognostic insight, they require extended follow-up and substantial resources. The present study aligns with a complementary paradigm, focusing on the early identification of individuals with cognitive impairment within a classification framework, with the aim of supporting clinical decision-making rather than replacing established diagnostic pathways.

Ultimately, the interpretation of predictive performance must be contextualized within the framework of data modality and algorithmic explainability. While logistic regression coefficients provide interpretable estimates of global feature effects and allow decomposition of the linear predictor into feature-wise contributions, such representations primarily reflect average effects and do not directly convey how feature contributions vary across individuals or across the range of observed values. In this context, SHAP provides complementary insights by enabling visualization of the distribution of feature effects, capturing inter-individual variability, and illustrating the relationship between feature values and their impact on model output within a unified framework. This complementary use of SHAP alongside inherently interpretable models has also been reported in recent studies applying SHAP to logistic regression models in clinical prediction settings [43]. This complementary role is further supported by the near-perfect agreement observed between SHAP-based and coefficient-based feature importance rankings in the present study.

Importantly, the robustness of these explainability findings was further explored through supplementary SHAP analyses conducted across black-box models (RF, XGBoost, SVM, and NB) based on the same consensus feature set (Supplementary Figure S2). These analyses demonstrated broadly consistent identification of key biomarkers, including tau, VEGF, and FAS, while Aβ42 was also frequently identified across different model architectures, suggesting that the observed feature importance patterns may not be limited to a single modeling approach. At the same time, the SHAP distributions of black-box models exhibited greater dispersion and less consistent directional patterns compared with the consensus-LR model, particularly for intermediate-ranked features, highlighting differences in interpretability and stability across model classes.

Consistent with these interpretability considerations, the SHAP-derived feature contributions identified in the present study appear broadly consistent with biological processes that have been associated with Alzheimer’s disease pathology and cognitive impairment. Core CSF biomarkers such as tau and Aβ42 are widely recognized indicators of neurodegeneration and amyloid-related pathology and represent key components of the ATN biomarker framework frequently used in Alzheimer’s disease research [8, 11]. In addition to these established markers, several proteins highlighted by the SHAP analysis-including NT-proBNP, pancreatic polypeptide, and IL-7-may reflect broader biological pathways that have been discussed in the literature in relation to neurodegenerative conditions, including vascular dysregulation, metabolic alterations, and inflammatory signaling. Interestingly, some biomarkers identified in the present analysis-particularly cystatin C and tau-have also been highlighted in previous machine learning studies based on the same CSF proteomic dataset, where they were reported as among the most informative markers for distinguishing cognitively impaired from cognitively normal individuals. Previous proteomic and machine learning-based studies have suggested that cerebrospinal fluid biomarker profiles may capture multiple biological processes potentially involved in cognitive impairment rather than representing a single dominant mechanism [10, 11, 21, 41]. Within this context, the partial concordance between SHAP-derived feature importance and previously reported biomarker associations may contribute to the biological interpretability of the model while remaining consistent with the exploratory nature of the present analysis. Furthermore, the absence of substantial multicollinearity in the consensus feature set (VIF < 5) supports the stability and reliability of the SHAP-based feature attributions, reducing the likelihood of misleading importance assignments due to correlated predictors.

Recent literature has seen the emergence of sophisticated imaging-based models and explainable ensemble frameworks [17, 19, 44] that achieve high diagnostic accuracy through multi-modal neuroimaging features. While these approaches provide granular insights into structural brain changes, they often necessitate high-cost infrastructure and specialized radiological expertise. Recent work applying machine learning models to distinguish individuals with mild cognitive impairment from cognitively normal controls has further demonstrated the capacity of biomarker-driven algorithms and ensemble learning strategies to capture complex interactions among clinical and biological variables [45]. In parallel, interpretable machine learning frameworks combined with explainability techniques such as SHAP have been employed to identify clinically meaningful markers associated with neurodegenerative processes and structural brain alterations, highlighting the growing emphasis on transparency and interpretability in clinical AI applications [46]. More broadly, recent evidence suggests that SHAP- and LIME-based approaches have become among the most widely adopted explainability tools in Alzheimer’s disease research, reflecting a broader shift toward interpretable and clinically contextualized machine-learning frameworks [47]. In addition, some recent studies have begun to extend model evaluation beyond discrimination by incorporating calibration and decision curve analysis, although such metrics remain less consistently reported than AUROC-based comparisons [48]. Within this evolving landscape, the present study extends prior work by not only applying SHAP-based interpretability, but also explicitly addressing feature-selection instability through a consensus framework integrating LASSO and Boruta, thereby providing a more robust and reproducible basis for biomarker identification.

In contrast, our model, which leverages biochemical CSF parameters, offers a distinct yet complementary vantage point. From a methodological perspective, the consensus-based feature selection strategy represents a key strength of this work, facilitating a more accessible biomarker profile for systematic clinical deployment. While many previous studies rely on a single selection technique-which may be sensitive to sampling variability and increase overfitting risk, as seen in recent models with limited selection pipelines [39, 49]-our study prioritizes feature stability and reproducibility by integrating multiple complementary approaches. This distinction is particularly relevant in the context of recent explainable machine learning studies, where interpretability is often enhanced through SHAP-based attribution, but predictor selection itself is still commonly driven by a single dominant pipeline or model-centric strategy [50]. This strategy explicitly contrasts with many existing explainable ML frameworks in which predictor selection is performed using a single pipeline or predefined feature subset, potentially limiting the robustness of biomarker identification across datasets. By systematically comparing LASSO-, Boruta-, and consensus-based feature sets, the present framework extends this line of research by explicitly addressing feature-selection instability rather than treating it as a secondary issue. Furthermore, the incorporation of explainable AI methods alongside calibration assessment and decision curve analysis enables a more comprehensive evaluation of model performance, linking predictive accuracy with probabilistic reliability and potential clinical utility. In addition, the utilization of an independent test set for final evaluation effectively mitigates ‘over-optimism’ and selection bias, aligning our framework with broader recommendations for methodological rigor and robustness in clinical AI development [1].

Beyond overall discriminative performance, calibration represents a critical yet often underreported aspect of clinical prediction models, particularly in the context of real-world decision-making. In the present study, the consensus-LR model demonstrated good calibration across the majority of the predicted probability range, with close agreement between predicted probabilities and observed outcome proportions. This finding supports the reliability of the model’s probabilistic outputs and suggests that the estimated probabilities of cognitive impairment may be cautiously interpreted in a clinical context. Minor deviations observed at the lower and upper extremes of the probability spectrum likely reflect data sparsity rather than systematic miscalibration, a phenomenon commonly encountered in moderately sized biomedical datasets. By explicitly evaluating calibration alongside discrimination and clinical utility, the present framework strengthens confidence in the model’s potential applicability for probability-informed clinical workflows, complementing its robust and interpretable design. Consistent with these findings, supplementary calibration analyses of the consensus-based alternative models (RF, XGBoost, SVM, NB, and the ensemble model) demonstrated greater deviations from the ideal calibration line, particularly within the intermediate predicted-probability range (Supplementary Figure S1). Among these models, XGBoost and the ensemble model showed relatively closer agreement with the ideal line, whereas RF, SVM, and NB exhibited greater departures, indicating less reliable probability estimation despite their competitive discriminative performance. These findings further support the use of logistic regression as the most clinically reliable model in the present framework, highlighting that strong overall predictive performance does not necessarily guarantee accurate and interpretable probability estimation. Although the exploratory ensemble model yielded favorable performance in several threshold-dependent metrics, its advantages were accompanied by increased structural complexity and reduced interpretability. In this context, the consensus-LR model remained preferable as the primary model because it provided a stronger balance between discrimination, calibration, transparency, and potential clinical usability.

Several limitations of this study should be acknowledged. First, the model was developed and evaluated within a single dataset, and external validation in independent cohorts is required to confirm generalizability across different populations and healthcare systems. This limitation is consistent with recent systematic evidence indicating that, despite encouraging predictive performance across dementia-related machine learning studies, many published models remain constrained by limited external validation, potential risk of bias, and restricted generalizability [51, 52]. In addition, although the consensus-based feature selection strategy aims to improve feature stability, the consistency of selected biomarkers across independent cohorts remains an important question that warrants further investigation in future external validation studies. Second, the use of cross-sectional data limits the ability to model disease progression or predict conversion from prodromal stages to Alzheimer’s disease. Longitudinal validation would provide additional insight into temporal stability and prognostic value.

Third, although the model emphasizes interpretability through clinically familiar variables, causal inferences cannot be drawn from the identified predictors. Finally, while the use of clinical variables together with CSF-derived biomarkers obtained through lumbar puncture enhances methodological scalability, it may not capture disease-specific biological processes detectable through advanced imaging or molecular biomarkers. Future work integrating these data sources in a tiered or hybrid framework may further improve predictive performance and the broader evaluation of potential clinical applicability.

## Conclusions

In conclusion, this study demonstrates that robust and interpretable ML models for the classification of Alzheimer’s disease**-**related cognitive impairment can be developed using clinical variables and CSF-derived biomarkers obtained through lumbar puncture. By emphasizing consensus-based feature selection, comprehensive performance evaluation-including discrimination, calibration, and decision curve analysis and model transparency, the proposed framework helps bridge the gap between methodological development and potential clinical applicability within a classification context. These findings suggest that pragmatic and clinically grounded artificial intelligence models may serve as a complementary analytical approach alongside biomarker and imaging-based strategies, as well as providing a foundation for future external validation and multimodal investigations in Alzheimer’s disease.

## Supplementary Information

Below is the link to the electronic supplementary material.

Supplementary Material 1

Supplementary Material 2

Supplementary Material 3

Supplementary Material 4

Supplementary Material 5

## Data Availability

The data analyzed in this study are publicly available and were originally reported by Craig-Schapiro et al. (20). The dataset is accessible through publicly available resources associated with the original publication. The analysis code supporting the findings of this study is available from the corresponding author, and a cleaned and well-documented version of the analysis pipeline can be provided to ensure transparency and reproducibility.

## Declarations

### Authors’ information

Fulden Cantaş Türkiş is an Assistant Professor at Muğla Sıtkı Koçman University, Faculty of Medicine, Department of Biostatistics. Her research interests include biostatistics, time-to-event data analysis, machine learning, artificial intelligence in healthcare, explainable artificial intelligence, predictive modeling, and statistical learning using real-world clinical data. She collaborates with multiple interdisciplinary research groups as the lead biostatistician and provides statistical consultancy for clinical and translational research projects.

## Abbreviations

AD: Alzheimer’s disease
AI: Artificial intelligence
APOE4: Apolipoprotein E ε4
ATN: Amyloid/Tau/Neurodegeneration biomarker framework
AUROC: Area under the receiver operating characteristic curve
AUPR: Area under the precision–recall curve
Aβ42: Amyloid beta 1–42
Boruta: Boruta feature selection algorithm
CDR: Clinical Dementia Rating
CI: Confidence interval
CSF: Cerebrospinal fluid
DCA: Decision curve analysis
ENA-78: Epithelial neutrophil-activating peptide 78
FAS: Fas cell surface death receptor
F1 score: Harmonic mean of precision and sensitivity
GRO-alpha: Growth-regulated oncogene alpha
IL-7: Interleukin 7
KIM-1: Kidney injury molecule 1
LASSO: Least absolute shrinkage and selection operator
LR: Elastic Net-regularized logistic regression
MCC: Matthews correlation coefficient
MIF: Macrophage migration inhibitory factor
ML: Machine learning
MMP-10: Matrix metalloproteinase 10
NB: Naive Bayes
NrCAM: Neuronal cell adhesion molecule
NT-proBNP: N-terminal pro–B-type natriuretic peptide
PAI-1: Plasminogen activator inhibitor 1
PAPP-A: Pregnancy-associated plasma protein A
PRROC: Precision–recall receiver operating characteristic
RF: Random Forest
RBF: Radial basis function
ROC: Receiver operating characteristic
SHAP: Shapley Additive Explanations
SVM: Support Vector Machine
TNF-RII: Tumor necrosis factor receptor II
TRAIL R3: TNF-related apoptosis-inducing ligand receptor 3
VCAM-1: Vascular cell adhesion molecule 1
VEGF: Vascular endothelial growth factor
XAI: Explainable artificial intelligence
XGBoost: Extreme Gradient Boosting
