# Supplementary material for: Explainable machine learning for the prediction of Alzheimer’s disease-related cognitive impairment: a consensus feature selection approach
Source: BMC Med Inform Decis Mak. 2026 May 29;26:284. doi: 10.1186/s12911-026-03585-z (PMC13417844; doi:10.1186/s12911-026-03585-z)
Supplement: Supplementary file 1 — Supplementary Material 1 [file 12911_2026_3585_MOESM1_ESM.docx]

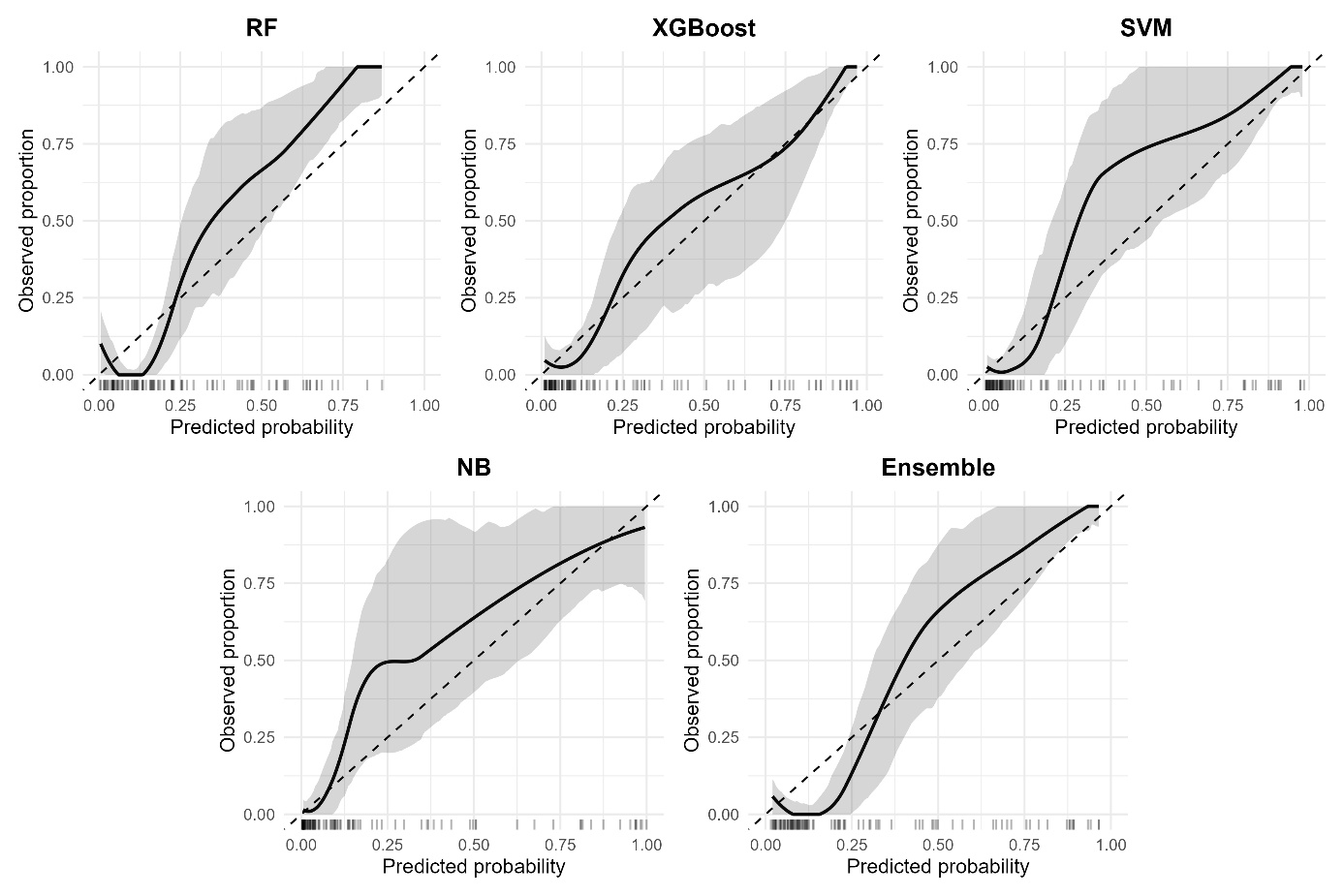


**Figure S1.** Calibration curves of machine learning models developed using the consensus data set.

*Panels show the calibration performance of random forest (RF), XGBoost, support vector machine (SVM), naive Bayes (NB), and the ensemble model evaluated on the independent test set. The solid line represents the loess-smoothed calibration curve, and the shaded area indicates the 95% confidence interval obtained via bootstrapping. The dashed diagonal line corresponds to perfect calibration. Rug plots along the x-axis illustrate the distribution of predicted probabilities.*
