# Supplementary material for: Explainable machine learning for the prediction of Alzheimer’s disease-related cognitive impairment: a consensus feature selection approach
Source: BMC Med Inform Decis Mak. 2026 May 29;26:284. doi: 10.1186/s12911-026-03585-z (PMC13417844; doi:10.1186/s12911-026-03585-z)
Supplement: Supplementary file 2 — Supplementary Material 2 [file 12911_2026_3585_MOESM2_ESM.docx]

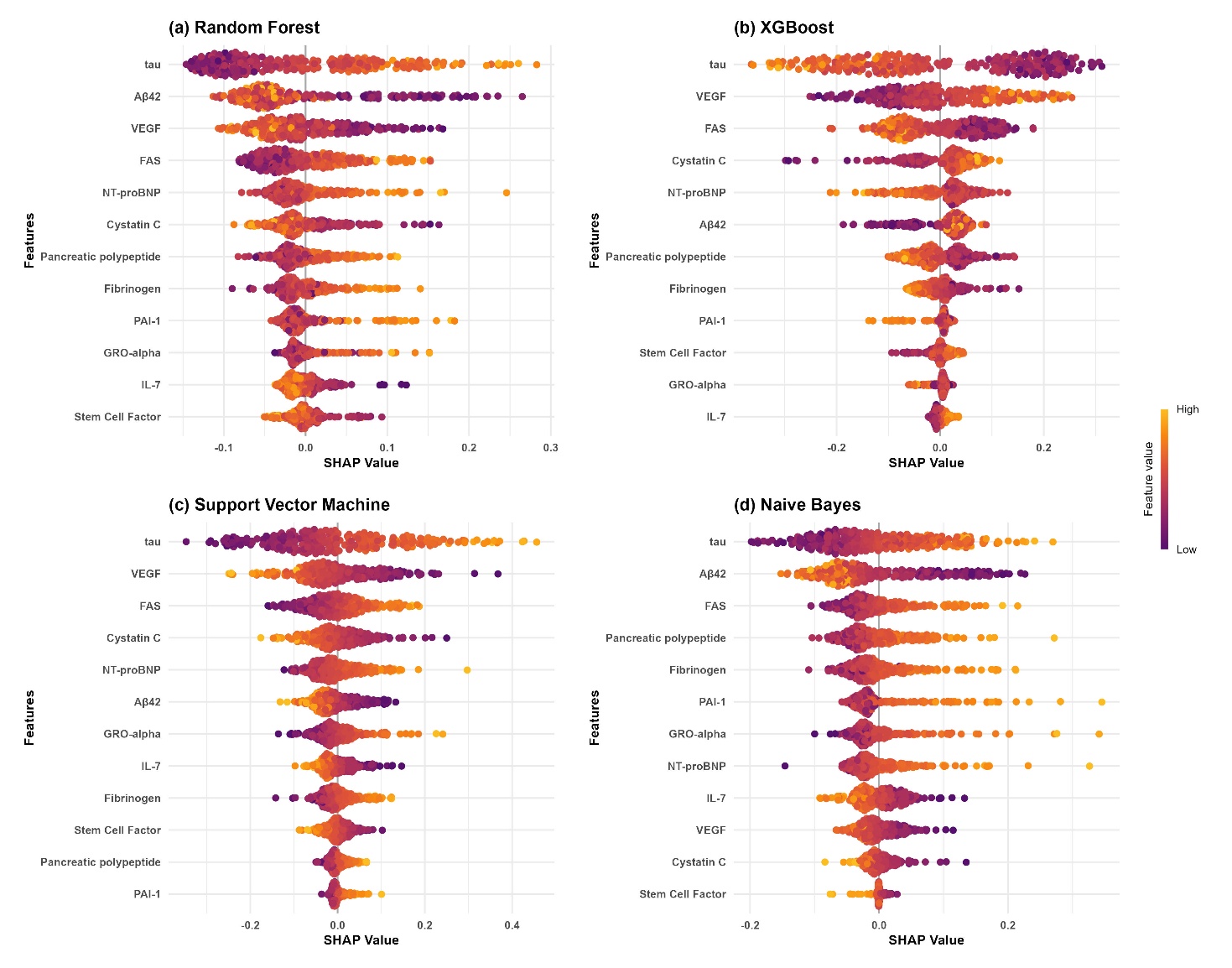


**Figure S2.** SHAP beeswarm plots of feature contributions across alternative machine learning models in consensus data set

*SHAP beeswarm plot illustrating the distribution of feature contributions across individual subjects. Each point represents a SHAP value for a given feature and individual. The horizontal position indicates the magnitude and direction of the feature’s contribution to the predicted probability of cognitive impairment, where positive values increase the predicted probability and negative values decrease it. Colors represent the corresponding feature values, ranging from low (purple) to high (yellow), enabling visualization of the relationship between feature magnitude and its impact on model predictions.*
