# Supplementary figures and images for: Explainable machine learning for the prediction of Alzheimer’s disease-related cognitive impairment: a consensus feature selection approach
Source: BMC Med Inform Decis Mak. 2026 May 29;26:284. doi: 10.1186/s12911-026-03585-z (PMC13417844; doi:10.1186/s12911-026-03585-z)

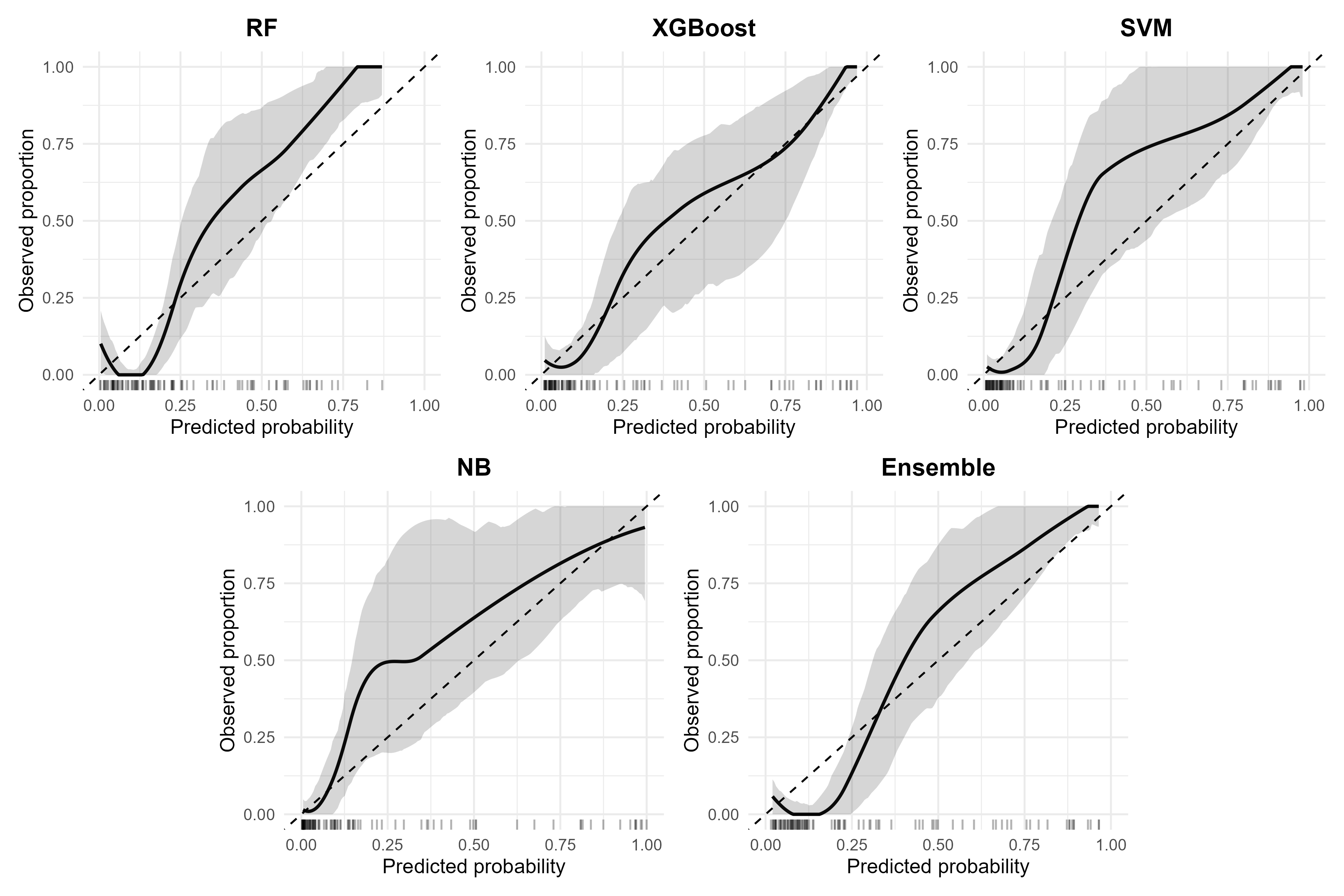

Supplement: Supplementary file 3 — Supplementary Material 3 [file 12911_2026_3585_MOESM3_ESM.jpeg]

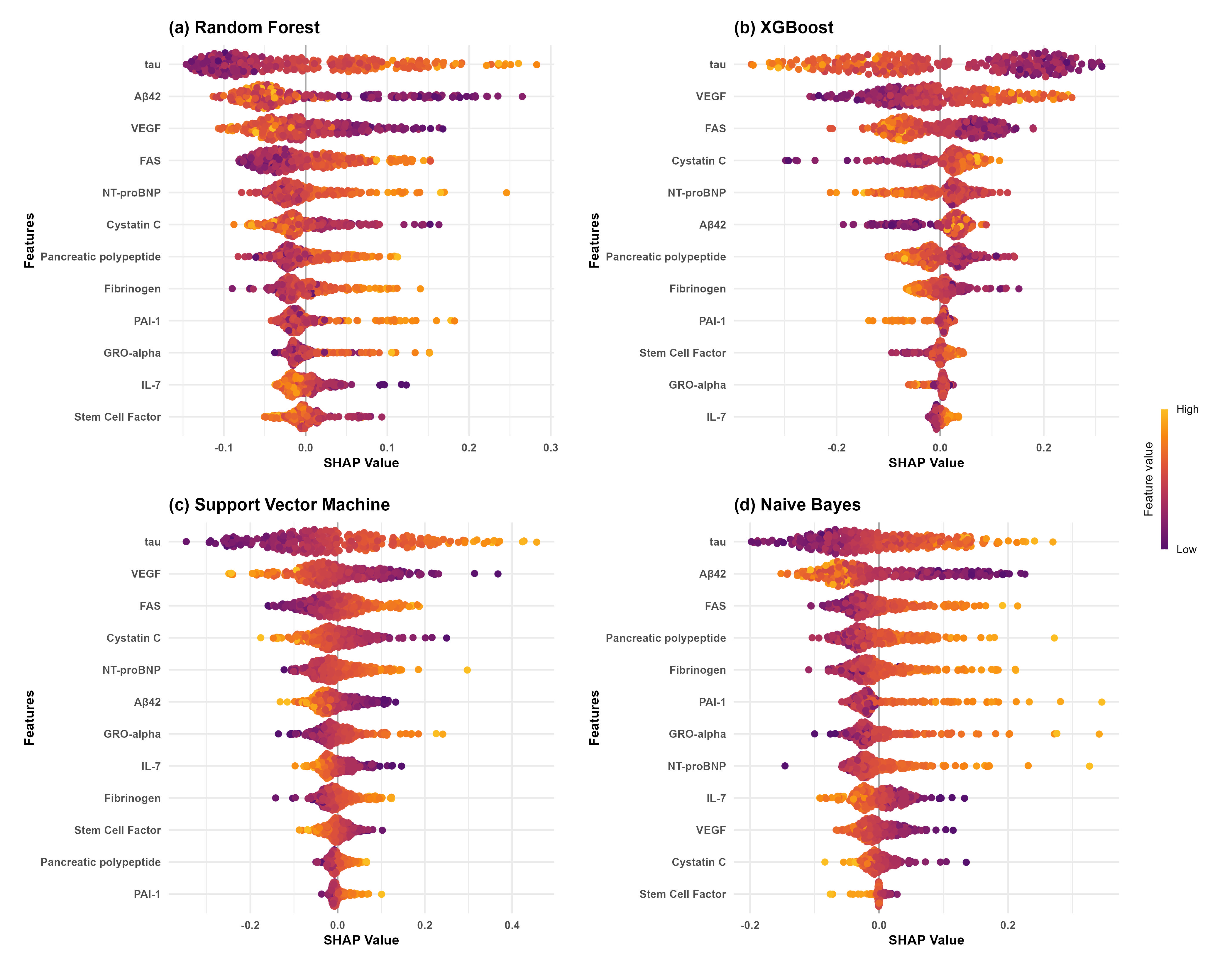

Supplement: Supplementary file 4 — Supplementary Material 4 [file 12911_2026_3585_MOESM4_ESM.jpg]
