# Supplementary material for: Explainable machine learning for the prediction of Alzheimer’s disease-related cognitive impairment: a consensus feature selection approach
Source: BMC Med Inform Decis Mak. 2026 May 29;26:284. doi: 10.1186/s12911-026-03585-z (PMC13417844; doi:10.1186/s12911-026-03585-z)
Supplement: Supplementary file 5 — Supplementary Material 5 [file 12911_2026_3585_MOESM5_ESM.docx]

**Table S1.** Final hyperparameter settings of machine learning models

| **Model** | **Hyperparameter** | **Baseline** | **LASSO data** | **Boruta data** | **Consensus data** |
| --- | --- | --- | --- | --- | --- |
| **LR** | alpha | 0.1 | 0.2 | 0.9 | 0.1 |
|  | lambda | 0.01559 | 0.01358 | 0.01358 | 0.03137 |
| **RF** | mtry | 2 | 2 | 9 | 3 |
|  | ntree | 500 | 500 | 500 | 500 |
|  | split rule | Gini | Gini | Gini | Gini |
| **XGBoost** | nrounds | 100 | 200 | 200 | 100 |
|  | max_depth | 2 | 4 | 4 | 3 |
|  | eta | 0.05 | 0.05 | 0.05 | 0.05 |
|  | gamma | 0 | 0 | 0 | 0 |
|  | colsample_bytree | 0.8 | 0.8 | 0.8 | 0.8 |
|  | min_child_weight | 1 | 1 | 1 | 1 |
|  | subsample | 0.8 | 0.8 | 0.8 | 0.8 |
| **SVM** | sigma | 0.01 | 0.01 | 0.01 | 0.01 |
|  | C | 1 | 4 | 2 | 4 |
| **NB** | fL | 0 | 0 | 0 | 0 |
|  | usekernel | TRUE | TRUE | TRUE | TRUE |
|  | adjust | 0.5 | 2 | 2 | 2 |

*LR: Elastic Net-regularized logistic regression; RF: Random Forest; XGBoost: Extreme Gradient Boosting; SVM: Support Vector Machine, NB: Naive Bayes.*

*Model-specific hyperparameters were optimized using ROC-based repeated cross-validation. Fixed parameters were kept constant across feature selection strategies to ensure comparability. The same tuning procedure was applied consistently across all feature sets, including the demographic-only baseline model, to ensure a fair comparison.*
